# Internalisation of RGD-Engineered Extracellular Vesicles by Glioblastoma Cells

**DOI:** 10.3390/biology11101483

**Published:** 2022-10-10

**Authors:** Dovydas Gečys, Arūnas Kazlauskas, Emilija Gečytė, Neringa Paužienė, Deimantė Kulakauskienė, Indrė Lukminaitė, Aistė Jekabsone

**Affiliations:** 1 Faculty of Pharmacy, Institute of Pharmaceutical Technologies, Lithuanian University of Health Sciences, LT-50162 Kaunas, Lithuania; 2 Laboratory of Molecular Cardiology, Institute of Cardiology, Lithuanian University of Health Sciences, LT-50162 Kaunas, Lithuania; 3 Laboratory of Molecular Neurooncology, Neuroscience Institute, Lithuanian University of Health Sciences, LT-50161 Kaunas, Lithuania; 4 Institute of Anatomy, Faculty of Medicine, Lithuanian University of Health Sciences, LT-44307 Kaunas, Lithuania; 5 Preclinical Research Laboratory for Medicinal Products, Institute of Cardiology, Lithuanian University of Health Sciences, LT-50162 Kaunas, Lithuania

**Keywords:** extracellular vesicles, EVs, exosomes, glioblastoma, targeted delivery

## Abstract

**Simple Summary:**

Glioblastoma multiforme (GBM) is the most aggressive and malignant type of central nervous system (CNS) tumour. Although several treatment options are available, patients generally succumb within 14 months after diagnosis. With the rapid progression of exosome bioengineering technologies, novel therapy opportunities are emerging for GBM treatment. The surface of GBM cells is characterised by the overexpression of transmembrane receptor integrins, which are essential for cell interactions with several proteins in the extracellular matrix. Therefore, integrin-binding drug delivery vehicles have been proposed as a potential strategy for glioblastoma therapy. Small extracellular vesicles possess several attractive characteristics for drug delivery: small size, biocompatibility, ability to cross the blood–brain barrier and capacity to be loaded with exogenous materials. Current bioengineering technologies further increase extracellular vesicle capabilities by loading them with anticancer drugs and incorporating targeting ligands. This study explored the capacity of Arginylglycylaspartic acid (RGD, or Arginine–Glycine–Aspartate)-polypeptide-engineered extracellular vesicles to internalise and deliver loaded cargo in GBM cells. The results demonstrate that introducing the RGD ligand to extracellular vesicles could significantly increase their internalisation by GBM cells and hence improve drug delivery efficacy.

**Abstract:**

Glioblastoma multiforme (GBM) is the most aggressive CNS tumour with no efficient treatment, partly due to the retention of anticancer drugs by the blood–brain barrier (BBB) and their insufficient concentration in tumour cells. Extracellular vesicles (EVs) are attractive drug carriers because of their biocompatibility and ability to cross the BBB. Additional efficiency can be achieved by adding GBM-cell-specific ligands. GBM cells overexpress integrins; thus, one of the most straightforward targeting strategies is to modify EVs with integrin-recognising molecules. This study investigated the therapeutic potential of genetically engineered EVs with elevated membrane levels of the integrin-binding peptide RGD (RGD-EVs) against GBM cells in vitro. For RGD-EV production, stable RGD-HEK 293FT cells were generated by using a pcDNA4/TO-Lamp2b-iRGD-HA expression vector and performing antibiotic-based selection. RGD-EVs were isolated from RGD-HEK 293FT-cell-conditioned medium and characterised by size (Zetasizer), specific markers (ELISA) and RGD expression (Western Blot). Internalisation by human GBM cells HROG36 and U87 MG and BJ-5ta human fibroblasts was assessed by fluorescent EV RNA labelling. The effect of doxorubicin-loaded RGD-EVs on GBM cells was evaluated by the metabolic PrestoBlue viability assay; functional GAPDH gene knockdown by RGD-EV-encapsulated siRNA was determined by RT-qPCR. RGD-EVs had 40% higher accumulation in GBM cells (but not in fibroblasts) and induced significantly stronger toxicity by loaded doxorubicin and GAPDH silencing by loaded siRNA compared to unmodified EVs. Thus, RGD modification substantially increases the specific delivery capacity of HEK 293FT-derived EVs to GBM cells.

## 1. Introduction

Since the first discovery of extracellular vesicles (EVs) 50 years ago [1], it has been proven that EVs play a significant role in critical cellular processes. Exosomes, a subset family of EVs, are membranous nano-vesicles (40–160 nm in diameter) with the natural capability to carry bioactive molecules, such as lipids, proteins and several RNA species [2,3,4]. Furthermore, it is well established that exosomes function as cell-to-cell messengers by transferring their cargo to recipient cells, resulting in alterations in cell homeostasis [5,6] and underlying their role in pathological states, such as cancer [7], neurodegenerative [8] and cardiovascular diseases [9] and psychological disorders [10]. 

The early concept of adapting exosomes as drug delivery vehicles was based on the essential characteristics of these vesicles: small size, biocompatibility [11], ability to cross the blood–brain barrier [12], and capacity to be loaded with exogenous materials [12,13,14]. The idea of exosome application as targeted drug delivery vehicles was further strengthened by biotechnology advancement, where cell-targeting capabilities were enhanced by using tissue-recognising molecules expressed on vesicle membranes. Alvarez-Erviti’s group was the first to show that the introduction of the rabies virus glycoprotein peptide (RVG) into primary-dendritic-cell-derived exosomes determines exosome accumulation in brain tissue after the intravenous administration of exosomes in mouse models [12]. Since then, in numerous studies, several tissue-targeting ligands have been used in exosome bioengineering, including RVG, RGD, Tlyp-1, chondrocyte-affinity peptide (CAP), E7-peptide and others [15]. Naturally, EVs are very heterogeneous, with considerable diversity based on their origin, morphology, size and cargo [16]. In addition, this diversity can be expanded even further by gene editing EV-secreting parental cells. 

The early concept of adapting exosomes as drug delivery vehicles was based on the essential characteristics of these vesicles: small size, biocompatibility [11], ability to cross the blood–brain barrier [12], and capacity to be loaded with exogenous materials [12,13,14]. The idea of exosome application as targeted drug delivery vehicles was further strengthened by biotechnology advancement, where cell-targeting capabilities were enhanced by using tissue-recognising molecules expressed on vesicle membranes. Alvarez-Erviti’s group was the first to show that the introduction of the peptide (RVG) into primary-dendritic-cell-derived exosomes determines exosome accumulation in brain tissue after the intravenous administration of exosomes in mouse models [12]. Since then, in numerous studies, several tissue-targeting ligands have been used in exosome bioengineering, including RVG, RGD, Tlyp-1, CAP peptide, E7-peptide and others [15]. Naturally, EVs are very heterogeneous, with considerable diversity based on their origin, morphology, size and cargo [16]. In addition, this diversity can be expanded even further by gene editing EV-secreting parental cells. 

Glioblastoma multiforme (GBM) is the most common and malignant type of tumour in the central nervous system, with one of the worst survival rates of all diseases worldwide. Due to its invasive capacity, heterogeneous histopathology and resistance to multiple chemotherapy drugs, this tumour has a high recurrence rate after treatment. Despite surgical and chemo/radiotherapy treatments, patients generally succumb within 14 months after diagnosis [16,17]. Studies focusing on targeted drug delivery could provide a much-needed breakthrough in GBM treatment. It has been established that the GBM cell surface is characterised by the overexpression of transmembrane receptors—integrins—that facilitate cell-to-cell and cell-to-extracellular matrix (ECM) adhesion, resulting in the increased invasiveness and survival of glioma cells [18]. Integrins, in particular, αvβ3 and αvβ5, have been proposed as attractive molecules for targeted therapies, mainly for their ability to recognise the RGD (Arginine–Glycine–Aspartate) polypeptide [19,20,21]. With current bioengineering technologies, various peptides can be introduced to EV surface membranes, enhancing particle binding to and internalisation by the cells of interest [12,22,23].

Several studies involving RGD-modified exosomes have yielded promising results in breast cancer [23] and alveolar basal epithelial cells derived from adenocarcinoma [24]. Recently, the capacity of the RGD peptide for GBM targeting was also investigated, including RGD-nanostructured lipid carriers [25], cRGD-installed micelles [26], RGD-poly(trimethylene carbonate)-based nanoparticles [27] and others. Additionally, Zhu and co-authors described post-isolation embryonic-stem-cell-derived EV modification with the c(RGDyK) motif by yielding improved GBM targeting in vitro and in vivo and enhancing the curative effects of EV-encapsulated paclitaxel when compared to the free drug alone [28]. 

Despite the simplicity of post-isolation EV surface modification, some disadvantages of such methods have been observed, including the possible alteration of membrane proteins, membrane damage or the decreased retention of EVs [13,29]. On the other hand, EV-producing cell engineering (pre-isolation modification of EVs) offers several advantages: the incorporation of a naturally occurring targeting peptide into the membrane during EV biogenesis, the stable overexpression of targeting molecules [13,30] or the loading of naturally occurring biological cargo [31]. 

This study aimed to establish a stable HEK 293FT cell line producing EVs with the RGD peptide, investigate the internalisation differences between RGD-modified extracellular vesicles (RGD-EVs) and native EVs by GBM cells in vitro, and examine the capacity of the bioengineered EVs to deliver the conventional anticancer drug doxorubicin and epigenetic modulator siRNA cargo to GBM cells. 

## 2. Materials and Methods

### 2.1. Experimental Design

**Figure 1:**
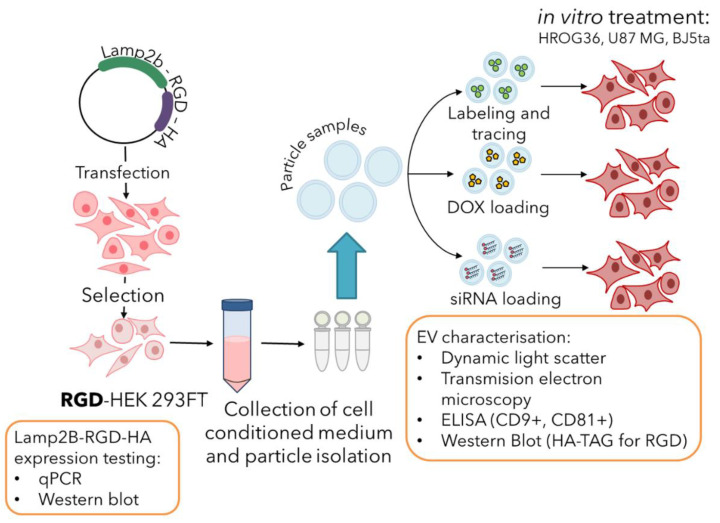
Experimental design of the study. The study was divided into three parts: (1) generation of a stable Lamp2b-RGD-HA HEK 293FT cell line; (2) isolation and characterisation of EVs; (3) downstream analysis, which includes EV internalisation studies, the delivery of doxorubicin hydrochloride (DOX) and the delivery of siRNA against the glyceraldehyde 3-phosphate dehydrogenase (GAPDH) gene.

The experimental design of the study is presented in Figure 1. A Lamp2b-RGD-HA cassette was designed, synthesised and inserted into the pcDNA4/TO plasmid. The stable Lamp2b-RGD-HA-expressing HEK 293FT cell line was generated by performing cell selection via zeocin resistance introduced by pcDNA4/TO. EVs were isolated from a cell-conditioned medium, characterised and prepared for in vitro cell culture introduction. The internalisation efficacy of EVs was assessed, and the ability to deliver externally loaded doxorubicin hydrochloride (DOX) and siRNA was evaluated in GBM cells.

### 2.2. Generation of Stable RGD-Expressing Cell Line RGD-HEK 293FT

The nucleotide sequence of the glycosylated Lamp2b-iRGD-HA fusion protein was synthesised according to Hung et al. [32] by BioCat GmbH (Heidelberg, Germany), with NheI cloning sites on both ends, replacing the RVG motif with the iRGD amino acid sequence (CRGDKGPDC), and inserted into a pUC57 vector. The sequence of the Lamp2b-iRGD-HA cassette was verified by Sanger sequencing. Afterward, the Lamp2b-iRGD-HA fragment was cloned into the pcDNA4/TO expression vector with ampicillin and zeocin (Thermo Fisher Scientific, Vilnius, Lithuania)) resistance using XbaI and amplified in competent *E. Coli* DH5α (Thermo Fisher Scientific, Vilnius, Lithuania) culture. Plasmid DNA extraction was performed using the GeneJET Plasmid DNA MiniPrep kit (Thermo Fisher Scientific, Vilnius, Lithuania). The quality of the pcDNA4/TO-Lamp2b-iRGD-HA construct was determined by agarose gel electrophoresis after digestion with the restriction enzyme BamHI (Thermo Fisher Scientific, Vilnius, Lithuania). 

To generate a stable RGD-HEK 293FT cell line, transfection of HEK 293FT cells with the pcDNA4/TO-Lamp2b-iRGD-HA vector was performed using jetPRIME transfection reagent (Polyplus, New York, NY, USA) under recommended conditions. Cell selection was conducted for four weeks using DMEM-F12-Glutamax cell medium (Thermo Fisher Scientific Gibco, The Netherlands) supplemented with 150 µg/mL zeocin, 10% foetal bovine serum (Thermo Fisher Scientific, Gibco, The Netherlands) and penicillin–streptomycin solution (10,000 IU/mL–10,000 μg/mL, Thermo Fisher Scientific, Gibco, The Netherlands). Lamp2b-iRGD-HA expression was confirmed by Western blot analysis of transfected and non-transfected HEK 293FT cell lysates (primary antibodies used: anti-human HA-tag (1:20,000; Invitrogen, Thermo Fisher Scientific, Bleiswijk, The Netherlands) and anti-human β-actin (1:2000; Thermo Fisher Scientific, Invitrogen, The Netherlands)) under denaturing conditions. Lamp2b-iRGD-HA mRNA expression was also determined using real-time quantitative PCR using Power SYBR Green PCR master mix (Applied Biosystems, Thermo Fisher Scientific, Bleiswijk, The Netherlands). The GAPDH gene was used as a reference. The primer sequences used in the study are provided in Table A1.

### 2.3. Extracellular Vesicle Isolation and Characterisation

The parental cell line HEK 293FT (Invitrogen, Thermo Fisher Scientific, The Netherlands) and stable RGD-HEK 293FT cells derived from it were cultivated in the conditions mentioned in the previous section. After reaching 80–90% confluency, the growth medium was replaced with a cell growth medium supplemented with EV-depleted FBS (Gibco, Thermo Fisher Scientific, Bleiswijk, The Netherlands) to minimise contamination with EVs present in the serum. After 48 h, cell-conditioned media were collected and passed through 0.22 µm PVDF filters to remove bigger particles. EVs from filtered cell-conditioned medium were isolated using Total Exosome Isolation Reagent (Invitrogen, Thermo Fisher Scientific, Bleiswijk, The Netherlands) according to the manufacturer’s instructions. Briefly, conditioned media were mixed with the isolation reagent at a ratio of 2:1. The mixtures were incubated at +4 ^○^C for 16 h and centrifuged at 10,000 g for 1 h at + 4 ^○^C. The resulting pellets were resuspended in 200 µL of PBS per 40 mL of the starting conditioned-medium volume.

The amount of total protein in isolated particle samples was determined with the Bradford assay (Sigma-Aldrich, Taufkirchen, Germany) using a Tecan Infinite 200 PRO plate reader (Tecan Austria GmbH, Grödig, Austria). Enzyme-Linked Immunosorbent Assays (ELISAs) were performed for common exosomal markers, tetraspanins CD81 and CD9, using sandwich-type commercial kits (MyBioSource, San Diego, CA, USA). The size distribution of particle samples was determined by nanoparticle tracking analysis (NTA) using a Nanosight DS300 analyser (Malvern PANalytical, Malvern, UK) according to the manufacturer’s protocol. 

The imaging of EV samples was performed using transmission electron microscopy (TEM). Frozen EV samples were thawed at room temperature, homogenised for 10 min with a 30 G needle and mixed in equal parts (1:1) with 4% paraformaldehyde solution. The final solution was applied on carbon-coated Formvar (FCFT200-Cu-50, Sigma Aldrich, Hoeilaart, Belgium) meshes and incubated at room temperature for 20 min. After incubation, the mesh was washed with 50 μL of PBS and fixed with 50 μL of 1% glutaraldehyde solution for 5 min. Next, fixated meshes were washed with distilled water and stained with 2% uranyl acetate for 5 min. After staining, the meshes were incubated with freshly prepared 2.25% methylcellulose and 2% uranyl acetate in a v/v ratio of 4:1 for 10 min at +4 °C. The prepared meshes were carefully dried on filter paper for 15 min and visualised using a Tecnai Bio Twin Spirit G2 (FEI, Eindhoven, The Netherlands) microscope at 120 kV voltage. Electron microscope images were taken with a bottom-mounted 16 MP TEM CCD camera (Eagle 4 K) employing TIA (FEI, Eindhoven, The Netherlands).

The presence of the recombinant Lamp2b-RGD-HA protein in isolated particle samples was determined by Western blot analysis using rabbit polyclonal anti-human HA antibody (Invitrogen, Thermo Fisher Scientific, Bleiswijk, The Netherlands). Briefly, 40 µg of total particle protein was loaded onto 10% SDS-PAGE gel and run for 2 h under denaturing conditions. Proteins were transferred onto a PVDF membrane and blocked in 5% BSA for 1 h at room temperature with gentle agitation. Afterward, a primary antibody (1:2000) in 5% BSA was added to the membrane and incubated overnight at +4 °C with gentle agitation. After incubation, a secondary anti-rabbit IgG-HRP antibody (1:50,000, Invitrogen, Thermo Fisher Scientific, Bleiswijk, The Netherlands) in 5% BSA was added and incubated at room temperature with gentle agitation. The membrane was visualised using the SuperSignal West Femto kit (Thermo Fisher Scientific, Vilnius, Lithuania) on a ChemiDoc XRS device (Bio-Rad, Hercules, CA, USA).

### 2.4. EV Uptake Assay 

Three cell lines—glioblastomas HROG36 (Cell Lines Service GmbH, Eppelheim, Germany) and U87 MG (European Collection of Cell Cultures (ECACC, Salisbury, UK), as well as human fibroblasts BJ-5ta (ATTC, Washington, DC, USA)—were analysed for exosome uptake. Cells (10,000 cells per dish) were seeded in 35 mm confocal Petri dishes. HROG36 cells were grown using DMEM-GlutaMAX cell growth medium supplemented with 10% foetal bovine serum and penicillin–streptomycin solution (10,000 IU/mL–10,000 μg/mL). U87 MG and fibroblast cells were cultivated in DMEM supplemented with 10% foetal bovine serum and penicillin–streptomycin solution (10,000 IU/mL–10,000 μg/mL). EVs (8 µg of total EV protein per dish) were labelled with SYTO RNASelect Green Fluorescent Cell Stain (Invitrogen, Thermo Fisher Scientific, Bleiswijk, The Netherlands) according to the manufacturer’s instructions. Labelled particle samples were cleaned of unincorporated dye using Exosome Spin Columns MW 3000 (Invitrogen, Thermo Fisher Scientific, Bleiswijk, The Netherlands). Sample fluorescence prior to and after the cleaning was measured with a Qubit 3.0 fluorometer (Life Technologies, Thermo Fisher Scientific, Bleiswijk, The Netherlands) (Appendix A, Figure A3). Particle samples were transferred onto cells. Cell imaging was performed on a Zeiss Axio Observer Z1 fluorescence imaging system (Zeiss, White Plains, NY, USA). Cell fluorescence intensity was quantified using ImageJ software (National Institutes of Health, Bethesda, MD, USA) [33]. 

### 2.5. Doxorubicin Loading 

DOX was loaded into EVs via electroporation using a MicroPulser electroporation device (Bio-Rad, Hercules, CA, USA) and Gene Pulser/MicroPulser Cuvettes (0.1 cm gap; Bio-Rad, Hercules, CA, USA). EVs (20 µg of total EV protein) were mixed with doxorubicin hydrochloride in electroporation buffer (10% glycerol with 500 mM sucrose [34]; buffer pH was adjusted to 7.0 using sodium hydrochloride). The total mixture volume was 90 μL, and 5 µg/mL doxorubicin hydrochloride for maximal loading of 1 µg EVs (1 µg refers to the total protein amount that was measured by the Bradford assay) was used. EVs were electroporated at 400 V with a double pulse. After electroporation, samples were incubated at 37 °C for 30 min. To remove unincorporated DOX, samples were cleaned using Exosome Spin Columns (MW 3000; Invitrogen, Thermo Fisher Scientific, Bleiswijk, The Netherlands) and filtered 3 times through Amicon 100 kDA filters (Merck, Darmstadt, Germany) (Appendix A, Figure A3). The DOX concentration in electroporated samples was calculated according to the standard curve of DOX fluorescence intensity. Electroporated DOX solution without particles in the sample was used as the negative control in further cell viability experiments. 

### 2.6. Cell Viability Assay

Cell viability was determined using PrestoBlue Cell Viability Reagent (Thermo Fisher Scientific, Invitrogen, Bleiswijk, The Netherlands). HROG, U87 MG and BJ-5ta (5000 cells per well) were seeded in 96-well plates and treated with electroporated EVs for 72 h. Samples without particles that underwent DOX electroporation and cleaning steps were used as a negative control. Viability testing was performed according to the manufacturer’s protocol. After 72 h, the growth medium was changed to a reaction mix composed of 90 μL of growth medium supplemented with 10 μL of PrestoBlue reagent (1:10 dilution) and incubated for an additional hour. The cell viability changes were detected by fluorescence intensity measurement (excitation 570 nm; emission 610 nm) using a Tecan Infinite 200 PRO plate reader (Tecan Austria GmbH, Grödig, Austria). Cell viability was expressed as a percentage of the values detected in untreated cell samples. 

### 2.7. siRNA Loading and Gene Expression Analysis

siRNA loading into particles was performed by lipofection using jetPRIME transfection reagents. Briefly, Cy5-labeled-GAPDH-siRNA (Invitrogen, Thermo Fisher Scientific, Bleiswijk, The Netherlands) was mixed with 200 µL of transfection buffer and 3 µL of transfection reagent and incubated for 10 min at room temperature. The transfection mix was added to 100 µL of EV sample and incubated for 30 min at room temperature. siRNA transfection solution mixed with PBS without particles was used as a negative control. Unincorporated micelles from EV samples were removed using Exosome Spin Columns, repeating the cleaning step 3 times. To determine sample purification and siRNA loading efficiency, Cy5 fluorescence was measured before and after cleaning on a Qubit 3.0 device (Appendix A, Figure A3). The purified transfection mix was added to 12-well plates (80 µL siRNA-loaded particle transfection mix/well) with 80,000 cells/well in 750 µL of growth medium. After 4 h, the cell medium was changed to remove unincorporated particles. Additionally, 10,000 cells per dish were seeded in 35 mm confocal Petri dishes and treated with siRNA-loaded particles for 4 h to visualise internalisation.

Total RNA from the cells was extracted using a PureLink RNA extraction mini kit (Invitrogen, Thermo Fisher Scientific, Bleiswijk, The Netherlands). Following RNA extraction, samples were treated with DNAse I (Thermo Fisher Scientific, Vilnius, Lithuania) according to the manufacturer’s manual and reverse transcribed using a High-Capacity Reverse transcription kit (Life Technologies, Thermo Fisher Scientific, Bleiswijk, The Netherlands). For real-time quantitative PCR using Power SYBR Green PCR master mix, b actin was used as an endogenous control. PCR was run on a 7900HT PCR system (Applied Biosystems, Foster City, CA, USA) under standard conditions. Changes in gene expression were calculated using the 2^−∆∆C^_T_ method [35] and are depicted in Log2 Fold Change form. Primer sequences are given in Table A1.

### 2.8. Statistical Analysis

All statistical analyses and visualisations were performed using GraphPad Prism 6 software (GraphPad Software Inc., San Diego, CA, USA). Data distribution was evaluated using the Shapiro–Wilk normality test. Quantitative differences between two groups were evaluated by Student’s *t* test.

## 3. Results

### 3.1. Lamp2b-RGD-HA Expression in RGD-HEK 293FT Cells and EVs

**Figure A1:**
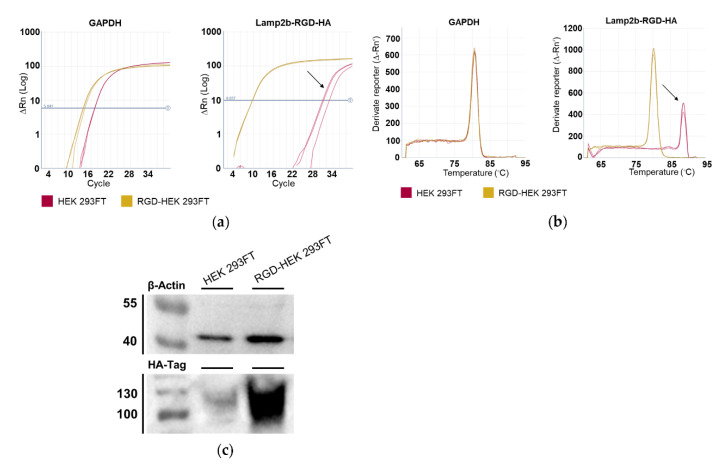
RT-qPCR and Western blot verification of HEK 293FT cells after zeocin-based resistance selection. (**a**) Amplification curves of Lamp2b-RGD-HA and GAPDH cDNA show retention of strong expression of Lamp2b-RGD-HA characterised by early amplification and detection. (**b**) Dissociation curves of amplified products. Arrow indicates nonspecific PCR product in parental HEK 293FT cDNA. (**c**) Western blot image of HEK 293FT and RGD-HEK 293FT cell lysates.

Stable RGD-HEK 293FT cells and transiently transfected HEK 293FT cells were analysed for Lamp2b-RGD-HA expression using RT-qPCR (Appendix A, Figure A1a) and Western Blot (Appendix A, Figure A1b) methods. Both tests confirmed the overexpression of Lamp2b-RGD-HA in RGD-HEK 293FT cells compared to parental HEK 293FT cells. 

**Figure 2:**
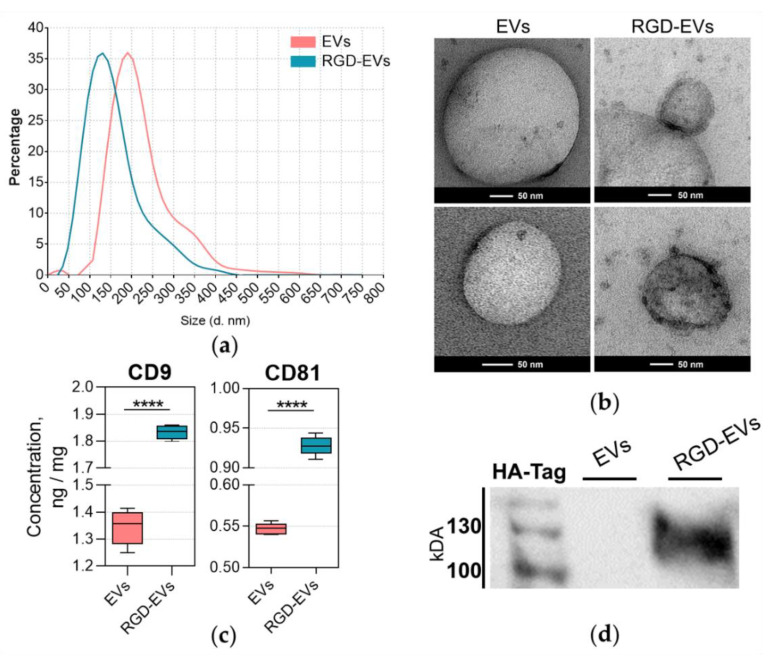
Characteristics of parental HEK 293FT and modified RGD-HEK 293FT EVs. (**a**) NTA results. The graphs show particle sizes ranging from 50 to 450 nm, with peaks of 120 for RGD-EVs and 180 nm for native EVs. (**b**) Representative TEM images. (**c**) ELISA confirmed the presence of tetraspanins CD9 and CD81 detected in EV isolates in HEK 293FT- and RGD-HEK 293FT-cell-conditioned media; **** *p* < 0.0001. (**d**) The Lamp2b-RGD-HA protein can be observed abundantly in the Western blot of RGD-EV particle samples but are absent in native EVs. Full blot images provided in Appendix A, Figure A2.

NTA of EVs purified from RGD-HEK 293FT and unmodified HEK 293FT cells (we termed them RDG-EVs and native EVs, respectively) showed the presence of particles ranging from 50 to 400 nm in size (Figure 2a). It was observed that RGD-EVs were, in general, smaller than native EVs. According to the results, the mean diameter of native EVs was 180 nm, whereas that of RGD-EVs was 120 nm. TEM imaging also confirmed the size difference, where most native particles were approximately 200 nm, and RGD-EVs appeared to be around 150 nm in diameter (Figure 2b). Most likely, such an EV size difference was induced by Lamp2 protein expression changes. 

Exosomal markers (tetraspanins CD81 and CD9) were detected in EV preparations by ELISA (Figure 2c) to indicate the presence of exosomes in purified EV samples. A substantial positive shift in CD9 and CD81 concentrations was observed in RGD-EV samples, which could indicate a transfection-related alteration in CD9 and CD81 secretion in RGD-HEK 293FT cells.

In addition, Western blot analysis using anti-HA-tag proteins confirmed the Lamp2b-RGD-HA presence in particle samples from RGD-HEK 293FT but not from HEK 293FT EV samples (Figure 2d). 

### 3.2. Tracing EV Uptake by HROG36, U87 MG and Human Fibroblast Cells

**Figure 3:**
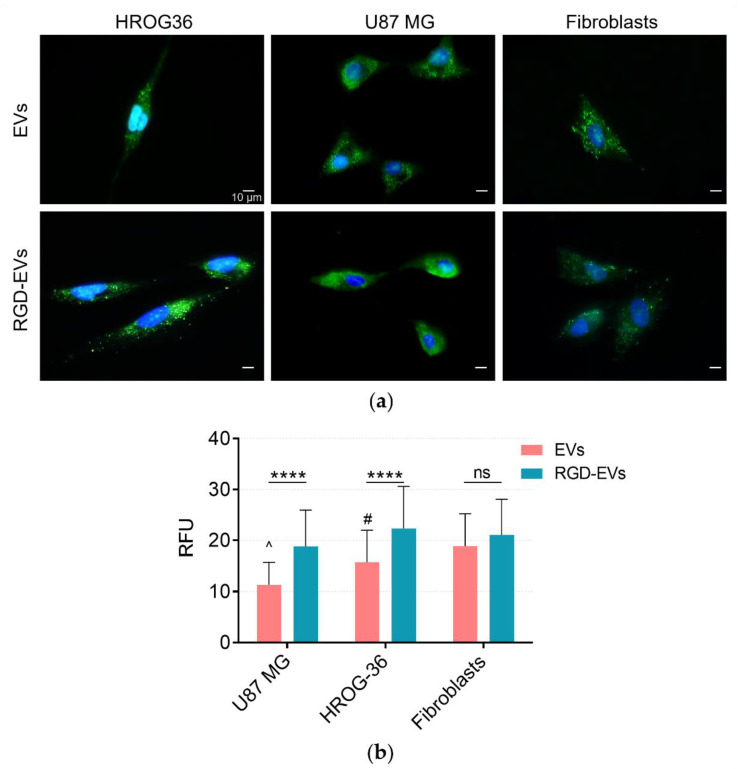
Tracing of EV uptake by HROG36, U87 MG and human fibroblast cells. (**a**) Representative fluorescence microscopy images of EV internalisation. Green fluorescence represents labelled RNA transferred to the cells by EVs. Blue fluorescence—Hoechst nuclear dye. (**b**) Quantitative evaluation of fluorescence intensity, which corresponds to amount of EVs internalised by the cells. Results demonstrate better internalisation of RGD-EVs compared to unmodified EVs by GBM and fibroblast cells following 4 h treatment. In contrast, no differences between the accumulation of RGD-EVs and native EVs were observed in fibroblasts. However, fibroblasts internalised native EVs faster compared to GBM cells; such a difference was not observed with RGD-EVs.; **** *p* < 0.0001 and ^#^ *p* < 0.05 when compared to fibroblasts; ^^^ *p* < 0.0001 when compared to fibroblasts; ns—not significant.

For the evaluation of EV uptake by GBM cells, EVs were labelled with the SYTO RNA Select green fluorescent cell stain, which is selective for RNA. HROG36, U87 MG and human fibroblast cells were incubated with stained EVs for 4 h, followed by fluorescent microscopy of treated cells. EVs are seen as green clusters in subcellular compartments within the cells, indicating that both native and RGD-modified EVs are internalised by GBM cells and human fibroblasts (Figure 3a). Further quantitative analysis of the images revealed differences in internalisation patterns between modified and native EVs (Figure 3b). The data show that RGD-EVs were taken up approximately 40% more effectively by GBM cells when compared to native HEK 293FT EVs. Moreover, this uptake difference was not observed in human fibroblasts. The results demonstrate that increasing the amount of RGD in EVs as a ligand for integrins on the GBM cell surface could dramatically improve the internalisation efficacy. 

### 3.3. DOX Delivery by Exosome-Like Particles

**Figure A3:**
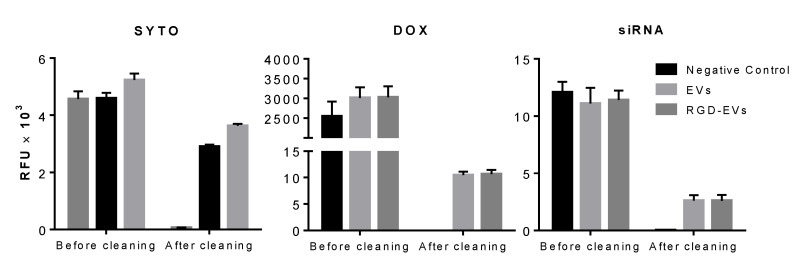
Fluorescence intensity measurements in EV during labelling and drug-loading procedures.

**Figure A4:**
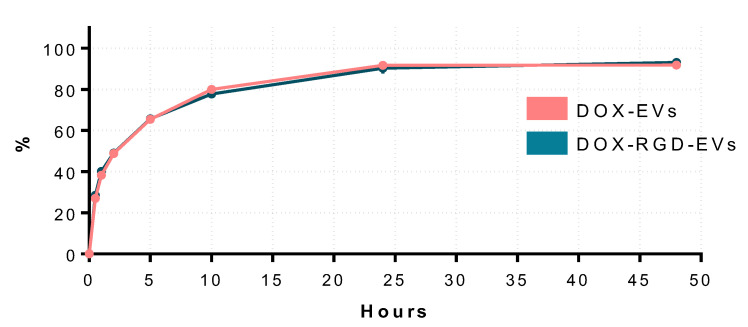
In Vitro DOX release profile. A 2 mL volume of EV-encapsulated DOX solution (130 ng/mL) was transferred to dialysis tubes (D977-100FT, Sigma Aldrich, Hoeilaart, Belgium), immersed in PBS at pH 7.4 and incubated at 37 °C. The release of EV-DOX was analysed at 7 time points (0.5, 1, 2, 5, 10, 24 and 48 h). Concentrations of DOX were measured by red fluorescence intensity and determined by calibration curve on Qubit 3.0 fluorimeter (Life Technologies, Thermo Fisher Scientific, Bleiswijk, The Netherlands). After performing drug release profile analysis of EV-DOX. No significant differences were observed between native and RGD-EVs. In PBS dialysate, the release rate of Exo-Dox was rapid and reached about 70% in the first 5 h.

Electroporation resulted in the loading of 1.25 ng of DOX per 1 µg of protein, which amounts to 0.03% loading efficiency and a total concentration of 625 ng/mL of DOX in loaded EV samples. During electroporation, a sample without particles was used in all steps to ensure that unincorporated DOX was being removed from EV samples during the cleaning step (Appendix A, Figure A3). These samples were used as a negative control in cell viability measurements. Additionally, after performing the encapsulated DOX release assay, no differences were detected between RGD-modified and native EVs (Appendix A, Figure A4). 

**Figure 4:**
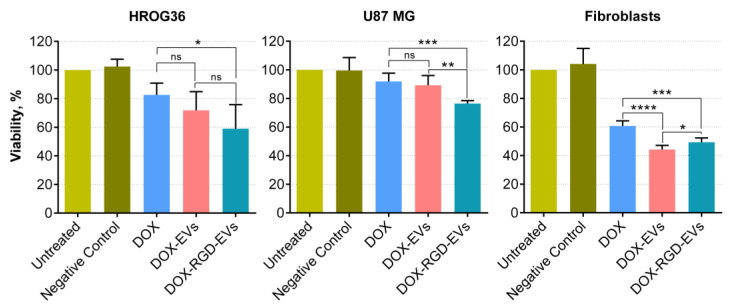
The effect of DOX-loaded EVs on GBM cell and fibroblast viability. Treatment with RGD-EV-encapsulated DOX caused a significant loss in GBM cell viability compared to treatment with free DOX and DOX loaded in RGD-EVs. Such differences between effects on cell viability were not observed in fibroblasts; in contrast, EV-encapsulated DOX decreased fibroblast viability to a significantly greater extent compared to free DOX. Negative Control means treatment with a sample that initially contained the DOX solution without EVs and underwent the same electroporation and cleaning steps as the EVs used for DOX loading. *n* = 5; * *p* > 0.05; ** *p* > 0.01; *** *p* < 0.001; **** *p* < 0.001.

Next, the efficiency of RGD-HEK 293FT EVs as drug carriers was evaluated by loading them with DOX and applying them to cultured GBM cells to examine the impact on their viability. HROG36 and U87 MG cells were treated with DOX-loaded EVs for 72 h using 12.5 ng/mL EV-encapsulated DOX and unincorporated DOX of the respective concentrations. DOX in RGD-EVs had a significantly stronger (by approximately 13%) adverse effect on the viability of both HROG36 and U87 MG cell lines compared to DOX in the native EVs. However, this tendency was not observed in fibroblasts, where DOX-loaded native EVs caused greater viability loss. (Figure 4). Notably, no statistically significant differences in the impact on GBM cell viability were observed between free DOX and unmodified-EV-encapsulated DOX. The same amount of DOX-free native EVs and RGD-EVs caused no negative changes in GBM and fibroblast viability after 72 h of treatment (data not shown).

### 3.4. siRNA Delivery to GBM and Fibroblast Cells by EVs 

**Figure 5:**
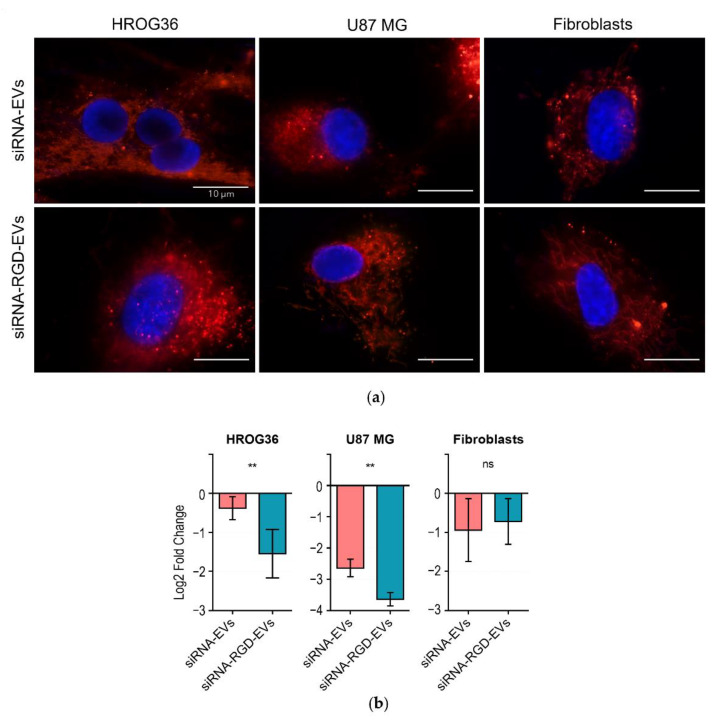
siRNA delivery to GBM and fibroblast cells. (**a**) Representative images of the internalisation of siRNA-loaded EVs by GBM and fibroblast cells. The accumulation of red fluorescent dye shows the distribution of siRNA molecules throughout the cells following 4 h of treatment. (**b**) RT-qPCR results summary. siRNA-loaded RGD-EVs caused significantly stronger GAPDH knockdown in GBM cells but not in fibroblasts. *n* = 6; ** *p* < 0.01.

Gene therapy is a promising approach in medicine, as it enables targeting specific disease-related genes with precision and personalised treatment options. However, such post-transcriptional gene modulators as siRNAs can be efficient only when applied in a carrier-encapsulated form to ensure the preservation and stability of the molecules in circulation. Therefore, in this study, we further investigated the possibility of loading EVs with exogenous siRNA molecules to evaluate targeted gene knockdown in cells and to determine differences in siRNA delivery between RGD-EVs and native EVs. Fluorescently labelled siRNA molecules were incorporated into EVs using lipofection and co-cultured with the recipient cells. In accordance with the above-described EV internalisation results (Figure 3), fluorescence microscopy images taken after 4 h of incubation exhibited the clear entry of siRNA-loaded EVs in both HROG36 and U87 cells, as well as human fibroblasts (Figure 5a). To investigate whether siRNA delivered by EVs would still be functional, a knockdown of the common endogenous control gene, glyceraldehyde 3-phosphate dehydrogenase (GAPDH), was used, as it is considered to be stably expressed among many cell lines, and its alteration in expression caused by siRNA can be accurately detected. EV-delivered siRNA efficiency was evaluated by monitoring GAPDH gene expression 48 h post-treatment with siRNA-loaded EVs (Figure 5b). The RT-qPCR results demonstrated that in HROG36 and U87 cells, siRNA-loaded RGD-EVs had significantly higher efficacy in GAPDH knockdown compared to siRNA carried by native EVs (HROG36 Log2FoldChange: −1.546 ± 0.65 vs. −0.379 ± 0.30, *p* = 0.0025; U87 Log2FoldChange: −3.637 ± 0.21 vs. −2.832 ± 0.39, *p* = 0.0014, Figure 5b).

In contrast, human fibroblast RT-qPCR data revealed no significant differences in GAPDH knockdown between samples treated with loaded RGD- and native EVs. These results support the hypothesis that increased effectiveness in the delivery can be achieved by using RGD as a targeting ligand for GBM cells in vitro.

## 4. Discussion

Glioblastoma multiforme (GBM) is one of the gliomas considered to be the most commonly occurring brain cell cancers [36]. Unfortunately, to this day, GBM remains incurable, as the current possible therapies, such as chemotherapy, radiotherapy, surgery and others, remain insufficient to remove GBM tumours [27]. Herein, we report the potential of bioengineered EVs in targeted drug delivery. We investigated the RGD peptide as a ligand to GBM cell surface proteins and integrins to enhance EV internalisation. 

The data demonstrate that modifying HEK 293FT cells with Lamp2b-RGD-HA results in RGD presence in RGD-EVS. Interestingly, the concentrations of tetraspanins CD9 and CD81 were significantly higher in RGD-EVs compared to native HEK 293FT EVs. A proteomic comparison of EVs performed by Kowal and co-authors concluded that tetraspanin enrichment in EV particle samples should be evaluated with caution, as EVs, including exosomes, produced by different cell types and even by the same cells may greatly vary, both qualitatively and quantitatively, in tetraspanin contents [37]. Moreover, a recent study showed that the Lamp2 protein family is involved in EV biogenesis and proteomic cargo packing, indicating that the overexpression of this protein could alter the proteomic profile of EVs, including the expression of tetraspanins [38].

Further experiments revealed that RGD-EVs were internalised more effectively when compared to native EVs. Additionally, GBM treatment with DOX incorporated in EVs displayed a more substantial suppressing effect on cell viability. It is important to emphasise that DOX incorporated into EVs had stronger negative effects on fibroblasts when compared to free DOX as well. Nevertheless, the difference between the effects of native and RGD-EVs on fibroblasts was different to those observed in the GBM group; native EVs had higher efficacy in reducing cell viability when compared to RGD-EVs. Moreover, RGD-EVs were more efficient in delivering GAPDH-suppressing siRNA. The data confirm that both native and RGD-modified EVs could carry exogenously added materials and enter GBM and fibroblast cells. qPCR analysis demonstrated that siRNA carried by RGD-EVs had a four times more potent effect on gene knockdown in HROG-36 cells when compared to native EVs. Less noticeable results were observed in U87-MG cells, where siRNA delivered by RGD-EVs had 1.3 times higher gene expression inhibition. The difference in expression inhibition between RGD-EVs and native EVs was not observed in fibroblasts. The GAPDH gene is widely used as an internal gene expression control in various experiments. In addition, Stanke and co-authors recently found that the high expression of glycolysis-controlling genes, including GAPDH, correlates with lower survival in GBM patients [39]. Thus, this siRNA could also potentially be tested as a novel therapeutic agent for GBM treatment.

Since EVs may enter cells in various ways, targeting peptides facilitate direct ligand–cell-surface-receptor binding, resulting in EV internalisation or membrane fusion and cargo release [40]. In addition to the aforementioned routes, EVs can be taken up by phagocytosis and endocytosis, which do not require specific EV–cell binding [41]. It is important to mention that variables affecting EV uptake are not entirely understood; however, cumulative evidence shows that EV internalisation is a strictly regulated process depending on the EV origin, type of cargo, route of administration or biological state of the recipient cell [42]. As there are points of uncertainty in the modes of EV internalisation, in vivo studies are necessary to determine the correct and desired biodistribution of particles during the development stages of targeted therapy. For example, Gong and colleagues showed that αvβ3 integrin-binding metalloproteinase-15-modified EVs are accumulated in breast cancer tumours. In contrast, unmodified EVs are primarily distributed in the mouse liver after the administration of EV solution via tail vein [23]. Another study reported that iRGD-modified exosomes successfully targeted B-cell lymphoma cells (characterised by αvβ3 overexpression) in mice by delivering siRNA molecules for cancer cell inhibition. The biodistribution analysis showed that iRGD-modified exosomes were mainly accumulated in the liver and kidney. Nevertheless, treatment with iRGD-modified exosomes resulted in stronger tumour inhibition compared to unmodified exosomes [43].

Similarly to our data, several studies have revealed that RGD-conjugated drugs have more significant curative effects on GBM cells when compared to unmodified compounds [44,45,46]. Furthermore, Xin and co-authors demonstrated that exosomes functionalised with RGD had favourable gastric-tumour-targeting properties [47]. In addition, a recent study reported that the targeted delivery of miR-484 via RGD-modified exosomes improves the survival time of tumour-bearing mice in an in vivo ovarian cancer model [48]. Furthermore, intravenously injected iRGD exosomes containing KRAS siRNA could reach targeted lung tumour tissues in vivo, resulting in the notable inhibition of tumour growth in mouse models [24]. With many excellent examples, RGD-binding integrin proteins have entered the spotlight in RGD-based targeted therapies. In a recent review, Ludwig and co-authors summarised that αvβ3, αvβ5, α5β1, αvβ6, αvβ8, α8β1, αIIbβ3 and αvβ1 have a significant role in cancer, participating in cellular processes such as tumour progression, cell migration, invasiveness, angiogenesis and others. GBM is known for its overexpression of αvβ3 and αvβ5, which are not expressed in normal brain tissue [20,49], thus enabling the exploration for targeted therapies.

According to our data and available literature sources, it can be concluded that RGD-EVs effectively strengthen binding to GBM cells; however, this method should not be considered to be only specific to GBM cells, as these EVs can be indirectly taken up by other cell types as well. These results coincide with other studies and provide supportive evidence of RGD modification prospects for research on potential targeted therapies. In addition, the establishment and characterisation of stable cell lines with targeting ligand overexpression could greatly contribute to EV studies, as they provide a complete platform of bioengineered EV sources. Despite that, currently, there are no clinical trials investigating RGD-EVs in general. Thus, introducing RGD as a ligand for integrin binding remains an interesting and promising strategy for targeted therapies.

## 5. Conclusions

RGD modification significantly increases the internalisation of HEK 293FT-derived EVs by GBM cells and, subsequently, improves EV-loaded DOX and siRNA delivery efficiency, making EVs with the RGD ligand a promising drug delivery strategy for GBM treatment.

## Figures and Tables

**Figure A2:**
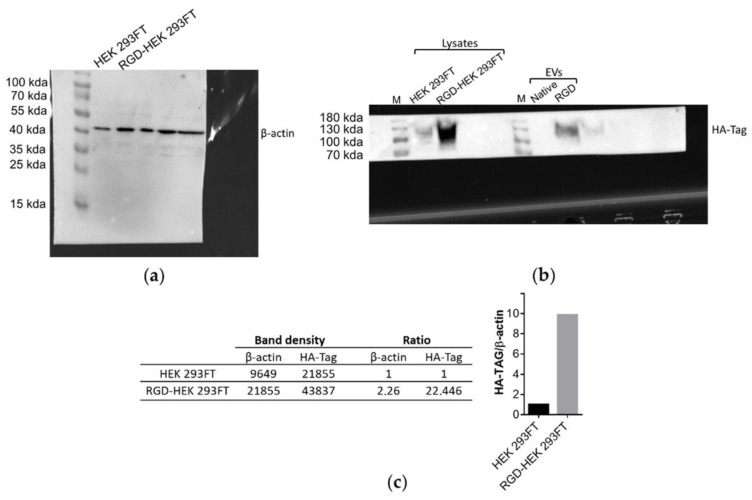
Raw blot images. (**a**) Blot image for HA-tag expression in cell lysates and EV samples. An overflow of RGD-HEK 293FT cell lysate sample from lane 3 to lane 2 can be seen, and slight spillage of RGD-EV sample can be seen from lane 3 to lane 4. (**b**) Blot image for b-actin expression in HEK 293FT and RGD-HEK 293FT cell lysate samples. (**c**) Densitometry analysis performed with ImageJ software for HEK 293FT and RGD-HEK 293FT cell lysates, normalised to b-actin.

## Data Availability

The raw data supporting the conclusions of this manuscript will be made available by the authors, without undue reservation, to any qualified researcher.

## Appendix A

**Table A1:** Primer sequences used in RT-qPCR.

Target	Sequence
β-Actin	F: AGAGCTACGAGCTGCCTGAC R: AGCACTGTGTTGGCGTACAG
GAPDH	F: TCAAGATCATCAGCAATGCCT R: CATGAGTCCCACGATACC
Lamp2b-RGD-HA	F: GGCGATAAAGGCCCGGATT R: CAGGAAAAGCCAGGTCCGAA
